# Semen Arousal: Its Prevalence, Relationship to HIV Risk Practices, and Predictors among Men Using the Internet to Find Male Partners for Unprotected Sex

**DOI:** 10.4172/2155-6113.1000546

**Published:** 2016-02-22

**Authors:** Hugh Klein

**Affiliations:** Kensington Research Institute, USA

**Keywords:** Men who have sex with men (MSM), Internet, Semen arousal, HIV risk practices

## Abstract

**Purpose:**

This paper examines the extent to which men who use the Internet to find other men for unprotected sex are aroused by semen. It also looks at the relationship between semen arousal and involvement in HIV risk practices, and the factors associated with higher levels of semen arousal.

**Methods:**

332 men who used any of 16 websites targeting unprotected sex completed 90-minute telephone interviews. Both quantitative and qualitative data were collected. A random sampling strategy was used. Semen arousal was assessed by four questions asking men how much they were turned on by the way that semen smelled, tasted, looked, and felt.

**Results:**

65.1 % of the men found at least one sensory aspect of semen to be “fairly” or “very” arousing, compared to 10.2% being “not very” or “not at all” aroused by all four sensory aspects of semen. Multivariate analysis revealed that semen arousal was related to greater involvement in HIV risk practices, even when the impact of other salient factors such as demographic characteristics, HIV serostatus, and psychological functioning was taken into account. Five factors were found to underlie greater levels of semen arousal: not being African American, self-identification as a sexual “bottom,” being better educated, being HIV-positive, and being more depressed.

**Conclusions:**

Being aroused by the sensory aspects of giving or receiving semen is commonplace amongst men in this high-risk population. Semen arousal was related closely to involvement in risk practices, indicating a need for HIV intervention programs to address this phenomenon in this population.

## Introduction

The risks of acquiring or transmitting HIV via unprotected anal sex have been well-documented and well-known for nearly 30 years now. Research based on populations of men who have sex with other men (MSM) has demonstrated that HIV/AIDS knowledge is generally in the moderate to high range, including a general understanding of the risks associated with engaging in unprotected anal sex [1–3]. Yet barebacking–that is, the act of engaging in intentional unprotected anal intercourse–persists in this population, with some recent evidence suggesting that, during the past 10 years or so, this behavior may have been on the increase [4,5]. In no small part, this helps to account for the stubbornly-steady rates of new HIV infections among American MSM during the past 10-20 years of the HIV/AIDS epidemic.

Many reasons help to explain the persistence of barebacking behaviors in this population. At the most rudimentary level, many MSM engage in unprotected anal sex because they perceive this type of sex to be “more natural” and to entail experiencing greater sexual sensations than comparable sex acts undertaken with the use of a condom [6,7]. For some men, engaging in unprotected anal sex is perceived to be more intimate and to foster a greater sense of connectedness with their sex partners than is the case when condoms are used [8,9]. In some instances, men avoid using condoms because of allergic reactions to the latex or polyurethane materials that are used to manufacture the condoms. Additionally, some men avoid using condoms because they do not like discussing HIV-related matters with their sex partners or do not feel comfortable broaching the subject of safer sex or feel confident in their ability to negotiate successfully for the use of condoms with new or existing partners [10,11]. Increasingly, anecdotal evidence also suggests that many MSM–especially younger MSM who did not live through the earlier years of the HIV/AIDS epidemic, when the media frequently provided images of sickly men who were suffering from and/or dying from the opportunistic infections associated with having HIV or AIDS–do not use condoms because they no longer fear becoming HIV-infected.

Almost always overlooked by HIV prevention professionals and interventionists working with MSM *and* by researchers whose studies focus on gay/bisexual male populations is the fact that many MSM do not use condoms simply because they do not want to use condoms. For many such men, condom use deprives them of something they enjoy and something that they find to be highly sexually arousing: the sensations associated with giving their semen to another man, receiving semen from another man, or sharing semen as part of the sex act(s) undertaken. Little has been written in the scientific literature specifically about semen and the meanings associated with giving, receiving, and/or exchanging semen in the context of male-to-male sex. This is the primary focus of the present paper.

Much of what has been written in the scholarly literature pertaining to the meanings associated with semen has come from either the feminist literature or from studies examining heterosexual couples and their (in) fertility issues [12,13]. A few notable exceptions do exist, though, with some authors specifically having addressed the topic of the meaning(s) of semen to various subpopulations of MSM. Prestage, Hurley, and Brown, conducted research with homosexually-active Australian men and found that nearly one-third of them reported semen exchange to be “very exciting.” Moreover, these researchers found that cum play was related inversely to condom attitudes and directly related to the extent to which men were sexual sensation seekers [14]. Schilder and colleagues conducted qualitative interviews with 12 HIV-positive and 12 HIV-negative gay men. They reported that swallowing semen was an integral part of many men’s (especially HIV-positive men) sex lives, and that receiving semen internally oftentimes was equated with increased sexual excitement, heightened sexual pleasure, and an increased sense of connectedness or intimacy with one’s sex partner. They also noted that many men (especially HIV-negative men) were afraid of or turned off by semen, fearing its capacity to transmit HIV or other sexually transmitted infections [15]. Examining the content of 281 online personal ads or postings focusing on the act of HIV-related gift giving (i.e., the process by which an HIV-infected person intentionally tries to find an HIV-negative person whom he can infect with HIV via sexual intercourse), Graydon reported that exchanging semen was construed as an act in which pleasure was mixed with pain, and that this was perceived to be a desirable combination. Moreover, some of the online messages pertaining to gift giving practices indicated a viewpoint that semen was “charged” or “hot” or “life giving,” which meant that it was construed as contributing to sexual satisfaction. Graydon also pointed out that giving semen to another man was considered to be a masculine activity [16]. Another study with a similar focus –namely, the content of three years worth of online postings focusing on gift giving and bug chasing (i.e., the process by which an HIV-negative man actively seeks HIV infection from a currently-HIV-positive man)–found that, similar to Graydon’s work, receiving semen as a bug chaser was construed as a feminizing activity whilst acting as the semen-providing gift giver was construed as a masculine behavior [16,17]. In this context, semen was viewed as being powerful and life-changing, with reproductive qualities (rather than producing babies in the usual heterosexual-reproductive meaning, however, the semen is seen as being capable of producing new gift givers). In a different study, another author also discussed the fact that giving or receiving semen oftentimes was perceived as having masculinizing or feminizing qualities [18]. In another article, Holmes and Warner conducted in-depth interviews about various aspects of semen exchange with 18 MSM in five major metropolitan areas in Canada and Europe [19]. As with Schilder et al.’s study, Holmes and Warner found that, for many men, semen exchange was necessary for a feeling of connectedness. Comparable findings were also reported by Gastaldo and colleagues [15,19,20]. Similar to Graydon’s work, Holmes and Warner; also noted that giving one’s semen to one’s sex partner oftentimes was construed as a gift, even among men who were HIV-negative [16,19].

Despite these few studies and their findings, what has not been addressed well in previously-published studies are the meanings that semen has for various subpopulations of MSM and the roles that semen plays in their sexual arousal and sexual behaviors. For so many MSM to continue to engage in unprotected sex of various types–not merely anal sex, but also oral sex, felching (the act of eating semen directly from another man’s anus after he received it during unprotected anal intercourse with one or more sex partners), snowballing (the act of sharing semen orally between partners, through deep kissing or drooling it back and forth into one another’s mouths), and other semen-exchanging sexual practices–suggests strongly that there is much more to the practice of semen delivery or semen exchange than merely disliking condoms, feeling less connected emotionally to one’s sex partner(s), disliking the loss of sexual sensation, and so forth. In the present paper, relying upon a sample of MSM who actively seek unprotected sex partners via the Internet, several research questions are examined; and all of them have been subject to very little previous scientific investigation: (1) To what extent do these men say that they are aroused by various sensory aspects of giving, receiving, or exchanging semen? (2) How, if at all, is their level of arousal from semen related to their involvement in HIV-related sexual risk practices? (3) What factors are associated with greater/lesser arousal from ejaculatory fluids?

## Methods

This paper draws from data that were collected in conjunction with The Bareback Project, a National Institute on Drug Abuse-funded study of men who use the Internet specifically to find other men with whom they can engage in unprotected sex. Some of the 16 websites from which the sample of 332 men were recruited catered exclusively to unprotected sex (e.g., Bareback.com, RawLoads.com). These sites accounted for 50.9% of the men subsequently recruited into the study. The other websites used did not cater to unprotected sex exclusively but did make it possible for site users to identify which men were looking for unprotected sex (e.g., Men4SexNow.com, Squirt.org). These sites supplied 49.1% of the men for the sample.

## Recruitment

A nationwide sample of men was derived, with random selection of participants being based on a combination of the first letter of the person’s online username, his race/ethnicity (as listed in his profile), and the day of recruitment. Each day, members of the research staff working on recruitment had three letters or numerals assigned to them for their use that day. These letters and numerals were assigned randomly and the entire alphabet and all numerals 0 through 9 were utilized in the randomization process. The first letter/numeral was restricted for use for recruiting Caucasian men only; the last two letters/numerals were to be used exclusively for recruiting men of color. This oversampling technique for racial minority group men was adopted so as to compensate for the fact that men of color, especially African-American men, are more difficult to recruit into research studies than their Caucasian counterparts are [21,22]. All letters of the alphabet and all numerals were used, randomly, for both Caucasian men and men of color, so that persons of all racial/ethnic groups would have an equal, random chance of having their profiles selected for potential participation in the study. In order for a particular person to be approached and asked to participate in the study, these letters/numerals had to correspond to the first letter/numeral of that individual’s profile and that person’s race/ethnicity, as stated in his profile, had to be a match for the Caucasian-versus-racial-minority-group-member designation on the daily randomization listing.

On recruitment sites where it was possible to know who was online at the time the recruiter was working, selection of potential study participants came from the pool of men who happened to be logged onto the site at the time when the recruiter was working. All men who were online at that time and whose profile name began with the appropriate letter/numeral were eligible to be approached. On recruitment sites where it was not possible to know who was online at the time the recruiter was working, ZIP codes were used to narrow down the pool of men who could be approached. To do this, in addition to the daily three letters/numerals that were assigned to each recruiter throughout the study, each day, ten five-digit numbers were also assigned to each recruiter (five to be used for Caucasian men, five to be used for men of color). These five-digit numbers were random number combinations and they were used in this study as proxies for ZIP codes. Recruiters entered the first five-digit number into the website’s ZIP code search field (which site users typically utilized to identify potential sex partners who resided within a specified radius from their residence), selected a five-mile radius, and then viewed the profile names of all men meeting those criteria who had logged onto that site within the previous 24 hours. Recruiters then reviewed the profiles of potentially-eligible men the letter/numeral match described above for men who were online at the time that recruiters were working.

Recruitment efforts were undertaken seven days a week, during all hours of the day and nighttime, variable from week to week throughout the duration of the project. This was done to maximize the representativeness of the final research sample, in recognition of the fact that different people use the Internet at different times.

## Participation

Initially, men were approached for participation either via instant message or email (much more commonly via email), depending upon the website used. Potential participants were provided with a brief overview of the study and informed consent-related information, and they were given the opportunity to ask questions about the study before deciding whether or not to participate. Interested men were scheduled for an interview soon after they expressed an interest in taking part in the study, typically within a few days. To maximize convenience for participants, interviews were conducted during all hours of the day and night, seven days a week, based on interviewer availability and participants’ preferences.

Participants in the study completed a one-time, confidential telephone interview addressing a wide array of topics, including degree of “outness,” perceived discrimination based on sexual orientation, general health practices, HIV testing history and serostatus, sexual practices (protected and unprotected) with partners met online and offline, risk-related preferences, risk-related hypothetical situations, substance use, drug-related problems, Internet usage, psychological functioning, childhood maltreatment experiences, HIV/AIDS knowledge, and basic demographic information. The questionnaire that was used was developed specifically for The Bareback Project. Many parts of the survey instrument were derived from standardized scales previously used and validated by other researchers. Interviews lasted an average of 69 minutes (median=63, *s.d.*=20.1, range=30–210). Participants who completed the interview were offered $35. Those who were comfortable supplying the interviewer with their name and mailing address were sent the $35 payment via regular mail. Men who wanted to remain more anonymous or “hidden” from the research team, or who wanted to receive their $35 more rapidly, were given the option of receiving that money via transfer of funds into an electronic PayPal account. Approval of the research protocol was given by the institutional review boards at Morgan State University, where the principal investigator and one of the research assistants were affiliated, and George Mason University, where the other research assistant was located.

## Qualitative data

Although the Bareback Project was primarily a quantitative study, qualitative data accompany the quantitative interviews for nearly three-quarters of the study participants (*n*=246). The qualitative data took the form of post-interview narrative summaries (what qualitative researchers often refer to as memos, or memoing; [23,24], in which the interviewers recorded personal observations and thoughts, direct quotes from the participants themselves, and contextual information that the interviewers believed would help to place the quantitative interview data into proper perspective. Each of the qualitative narrative summaries was anywhere from one-half of one page to three pages in length, depending upon how talkative the study participant was during and/or after the interview, and upon how much useful information the interviewer felt should be recorded at the interview’s conclusion. The idea underlying the memoing process was to capture information that otherwise would have been lost if the study had relied solely upon the quantitative information contained in the survey instrument–information that, hopefully, could be used to illuminate and inform major study findings. It was from these qualitative data that some of the interpretations of the findings obtained herein (presented in the Discussion section) were derived. Please note: In the present paper, rather than presenting fully-analyzed qualitative data, the qualitative data are utilized primarily to help understand and interpret the quantitative findings. Thus, the qualitative data are put to use in the Discussion section and appear only in that portion of this article.

## Measures Used

The principal measures of interest in these analyses were four questions asking men about the extent to which they were sexually aroused by various aspects of semen (alternately referred to as “semen arousal” and “eroticizing ejaculatory fluids” throughout the remainder of this paper). The topic was introduced in the questionnaire as follows: “In these next few questions, I am going to ask you about semen, or what many men refer to as cum.” Then respondents were asked: (1) “How much are you personally turned on by the taste of cum?” (2) “How much are you personally turned on by the smell of cum?” (3) “How much are you personally turned on by the feel of cum, either on your face or body or penis?” and (4) “How much are you personally turned on by the sight of cum, either on yourself or on someone you are having sex with?” Responses were scored on a five-point ordinal scale, ranging from “not turned on by it at all” (score=0) to “very turned on by it” (score=4). Subsequently, these items were combined into a single scale measure that evaluated their overall level of arousal by semen, with higher scores indicating greater arousal. Scores on the scale ranged from 0 to 16 (mean=9.17, *s.d.*=4.76), and the scale was found to be reliable (Cronbach’s alpha=0.84).

Several sexual risk behavior measures, all using a past-30-days time frame of reference, were examined as dependent variables in conjunction with Research Question #2. These included: the proportion of all sex acts involving the use of condoms (continuous), the proportion of anal sex acts involving the use of condoms (continuous), the proportion of oral sex acts involving the use of condoms (continuous), the proportion of all sex acts involving internal ejaculation (continuous), the proportion of anal sex acts involving internal ejaculation (continuous), the proportion of oral sex acts involving internal ejaculation (continuous), the number of sex partners (continuous), the number of times engaging in anonymous sex (continuous), and whether or not the person engaged in any multiple-partner sex acts (dichotomous).

To examine Research Question #3, consistent with the Syndemics Theory paradigm that guided *The Bareback Project* (for further details about this paradigm and its applicability to HIV risk practices among MSM, readers are encouraged to consult Klein [25], Mustanski et al. [26] and Stall et al. [27], five types of variables were used: demographic characteristics, substance use/abuse measures, items assessing psychological or psychosocial functioning, preferences for how to have sex, and childhood maltreatment experiences. Demographic characteristics examined included: age (continuous), race (three separate dichotomous measures, indicating Caucasian versus men of color, African American versus all others, and Latino versus all others), educational attainment (dichotomous, comparing men with no more than a high school education to those with at least some college experience), relationship status (dichotomous, comparing single men to those who were married or “involved” in a relationship), sexual orientation (dichotomous, comparing men self-identifying as gay to all others), sexual role/position preference (categorical, comparing “tops,” “bottoms,” and “versatile” men), HIV serostatus (dichotomous, comparing HIV-positive men to all others), and degree of urbanization where the men resided (continuous).

Four substance use/abuse measures were examined. These were: the total amount of illegal drug use reported during the past 30 days (continuous, summing quantity * frequency of use for nine different types of illegal drugs), the amount of methamphetamine use during the past 30 days (continuous), the number of drug-related problems experienced during the past year (continuous scale measure, Kuder-Richardson_20_=0.87), and the number of drug-related problems experienced during the past 30 days (continuous scale measure, Kuder-Richardson_20_=0.79).

Six scale measures were used to assess various aspects of psychological and psychosocial functioning. Self-esteem was assessed with Rosenberg’s well-known ten-item scale (Cronbach’s alpha=0.89) [28]. Depression was assessed using the Center for Epidemiologic Studies 20-item *CES-D* scale (Radloff, 1977; Cronbach’s alpha=0.93). Current life satisfaction was measured using five items taken from Diener et al.’s *Satisfaction with Life* scale (Cronbach’s alpha=0.83) [29,30]. Six items from Scheier and Carver’s *Life Orientation Test–Revised* were used to assess optimism about the future (Cronbach’s alpha=0.78) [31]. Impulsiveness was examined via 15 items derived from the work done by Von Diemen and colleagues based on the *Barratt Impulsiveness Scale* (Cronbach’s alpha=0.76) [32]. HIV information burnout was assessed via seven questions devised specifically for use with *The Bareback Project* (Cronbach’s alpha=0.55).

Respondents’ sexual safety matters and preferences for how to have sex were assessed via a series of questions asking men whether or not they liked to engage in anonymous sex (yes/no), how much they enjoyed having sex that was “wild” or “uninhibited” (by their definition; continuous), how much they enjoyed having sex in public places (continuous), how much they enjoyed having sex that was long-lasting (by their definition; continuous), the extent to which they preferred having sex that was rough versus gentle (by their definition; continuous), and how much they enjoyed having sex in venues such as bathhouses or sex clubs (continuous). Also included here are scale measures assessing condom use self-efficacy [which was examined via 13 items derived from the work done by Brafford and Beck (Cronbach’s alpha=0.86)], overall attitudes toward condom use [measured by 17 items adapted from Brown (Cronbach’s alpha=0.91)], and level of knowledge about HIV/AIDS transmission [assessed via 15 items adapted from the work done by Carey, Morrison-Beedy, and Johnson (Kuder-Richardson_20_=0.76)] [33,34].

Childhood maltreatment experiences were measured using the *Childhood Trauma Questionnaire* [35]. Six measures, all of which asked men about their experiences prior to the age of 18, were used: sexual abuse (Cronbach’s alpha=0.93), physical abuse (Cronbach’s alpha=0.85), emotional abuse (Cronbach’s alpha=0.89), physical neglect (Cronbach’s alpha=0.71), emotional neglect (Cronbach’s alpha=0.93), and total amount of childhood maltreatment experienced (Cronbach’s alpha=0.94).

## Analysis

Research Question #1 (pertaining to the extent to which respondents reported being aroused by various aspects of semen during sex) was examined via descriptive statistics. Research Question #2 (which pertains to the relationship between eroticizing ejaculatory fluids and involvement in risky sexual behaviors) and Research Question #3 (which focuses on the factors associated with greater/lesser eroticism from semen) were undertaken in similar fashion to one another, each involving two steps. First, bivariate relationships were assessed for each of the independent variables outlined above and the dependent measure(s) in question. Whenever the independent measure was dichotomous (e.g., sexual orientation, HIV-positive serostatus), Student’s *t* tests were used. Whenever the independent variable was continuous (e.g., amount of illegal drug use, self-esteem level), correlation coefficients (Pearson’s *r*) were computed. Then, all items found to be related either significantly (*p*<0.05) or marginally (0.10>*p*>0.05) to the dependent variable were entered into a multivariate equation, and then removed in stepwise fashion until a best fit model containing only statistically-significant measures remained.

## Results

### Sample description

In total, 332 men participated in the study. They ranged in age from 18 to 72 (mean=43.7, *s.d.*=11.2, median=43.2). Racially, the sample is a fairly close approximation of the American population [36], with 74.1% being Caucasian, 9.0% each being African American and Latino, 5.1% self-identifying as biracial or multiracial, 2.4% being Asian, and 0.3% being Native American. The large majority of the men (89.5%) considered themselves to be gay and almost all of the rest (10.2%) said they were bisexual. On balance, men participating in *The Bareback Project* were fairly well-educated. About 1 man in 7 (14.5%) had completed no more than high school; 34.3% had some college experience without earning a college degree; 28.9% had a bachelor’s degree; and 22.3% were educated beyond the bachelor’s level. Consistent with the demography of the U.S. population [37], 28.0% of the men lived in rural or low-density population areas (fewer than 500 persons per square mile), 23.5% lived in urban or higher-density population areas (more than 5,000 persons per square mile), with most of the latter group (17.2% of the sample) living in very high density population areas (more than 10,000 persons per square mile). Slightly more than one-half of the men (59.0%) reported being HIV-positive; most of the rest (38.6%) were HIV-negative.

### Research question #1 – arousal from ejaculatory fluids

Table 1 presents a summary of the data for the questions assessing the extent to which study participants experienced arousal from the various sensory aspects of giving, receiving, or exchanging semen. Study participants were least likely to be turned on sexually by the smell of ejaculatory fluids, with 30.4% of the men saying that they found this sensory aspect of semen to be “fairly” or “very” arousing compared to 37.3% of the men saying that they found it “not at all” or “not very” exciting to them sexually. When it comes to the way that semen tastes, men were slightly more likely to say that they found this “fairly” or “very” sexually arousing (41.1%) than they were to say that they did not find this “at all” arousing or “not very” arousing (34.1%). More than one-half of the respondents (52.9%) said that they found the way that semen feels to be “fairly” or “very” sexually exciting, compared to 30.1% who said that they found the way semen feels to be either “not at all” or “not very” arousing. Men were most turned on by the way that semen looks, either on themselves or on their sex partners. Nearly two-thirds of the men in the study (64.6%) reported that seeing ejaculatory fluids on themselves or their partner)s) was “fairly” or “very” sexually exciting for them, compared to only 15.6% who said that they found this to be “not at all” or “not very” exciting. On the overall scale measure, mean scores on the 0–16 scale were 9.17 (*SD*=4.76), with 37.3% of the study participants averaging responses in the “fairly” to “very” high range with regard to how erotic they found ejaculatory fluids to be. Looked at another way, nearly two-thirds of the men who took part in the study (65.1%) said that they found at least one aspect of semen–either its flavor, its smell, the way it feels, and/or the way it looks on someone’s face or body–to be “fairly” or “very” erotic. This compares to a mere 10.2% of the men who said that they found all four of these sensory aspects of semen to be “not at all” or “not very” arousing.

### Research question #2 – relationship between semen arousal and involvement in sexual risk behaviors

The extent to which men said that they were turned on by various sensory aspects of semen was related inconsistently to their involvement in specific sexual risk behaviors. In all instances in which a statistically-significant relationship was identified, it was always in the direction of greater semen arousal being associated with greater involvement in the risk behavior. This was true for the proportion of all sex acts involving internal ejaculation (*r*=0.18, *p*=0.002), the proportion of oral sex acts involving internal ejaculation (*r*=0.23, *p*<0.001), whether or not the person had engaged in any multiple-partner sexual encounters (*OR*=1.10, *CI*_95_=1.05-1.15, *p*<0.001), and whether or not the person had engaged in any anonymous sex encounters (*OR*=1.07, *CI*_95_=1.02-1.12, *p*=0.005). No associations were found between eroticizing ejaculatory fluids and the overall proportion of sex acts involving the use of protection (*r*=0.05, *p*=0.426), the proportion of oral sex acts involving the use of protection (*r*=0.08, *p*=0.153), the proportion of anal sex acts involving the use of condoms (*r*=0.03, *p*=0.595), the proportion of anal sex acts involving internal ejaculation (*r*=0.04, *p*=0.477), the number of recent sex partners (*r*=0.08, *p*=0.168), or the number of times engaging in anonymous sex (*r*=0.05, *p*=0.339).

Table 2 provides summary information for multivariate analyses undertaken to determine whether or not eroticizing ejaculatory fluids remained a consequential variable even when the effects of other influential measures were taken into account. (data not shown; for additional details about these bivariate analyses, please contact the author.) Only the four sexual behavior measures for which a statistically-significant bivariate relationship are included in this table. As the top line of Table 2 shows, for three of the four outcome measures in question, the extent to which men were turned on by semen was a key predictor. For the fourth measure (i.e., whether or not the person had engaged in any anonymous sex), this variable was found to be marginally statistically significant.

### Research question #3 – factors associated with eroticizing ejaculatory fluids

Of the various demographic variables examined, bivariate analyses revealed four that were related to the extent to which men eroticized ejaculatory fluids. The first of these was race, with African Americans reporting significantly lower levels of arousal from semen than their non-African American counterparts (6.37 versus 9.45; *t*=3.44, *p*<0.001). The second variable here was sexual role identity, with greater semen arousal being reported by men who self-identified as sexual “bottoms” than among men who self-identified as sexual “tops” (*r*=0.17, *p*=0.002). Third, men who said that they had no more than a high school education scored lower on the semen arousal scale than their better-educated counterparts did (7.48 versus 9.46; *t*=2.69, *p*=0.008). Fourth, HIV-positive men were more turned on by ejaculatory fluids than their HIV-negative or HIV serostatus-unknown counterparts were (9.65 versus 8.49; *t*=2.20, *p*=0.029).

Only one of the substance use/abuse measures examined was found to be associated with the extent to which men eroticized ejaculatory fluids. The more drug problems that men had experienced during the month prior to interview, the more they tended to say that they were turned on by different sensory aspects of semen (*r*=0.12, *p*=0.023).

Bivariate analyses showed that three of the psychological / psychosocial functioning measures were related to men’s levels of arousal from semen. Men with higher self-esteem tended to report lower levels of semen arousal than their lower-self-esteem counterparts (*r*=0.14, *p*=0.013). Moreover, the more men experienced depressive symptoms, the more they tended to be turned on by the various sensory aspects of ejaculatory fluids (*r*=0.18, *p*=0.001). Additionally, the more optimistic men were about the future, the less aroused they tended to be by semen (*r*=0.13, *p*=0.022).

Several of the items pertaining to men’s preferences for how they most liked to have sex were also found to be related to their levels of eroticism from ejaculatory fluids. First, men who said that they did not care whether potential sex partners were HIV-positive or HIV-negative scored higher on the semen arousal scale than men who indicated a specific HIV serostatus preference in their sex partners (10.06 versus 8.78, *t*=2.24, *p*=0.026). Second, higher levels of condom use self-efficacy were associated with somewhat levels of semen arousal (*r*=0.10, *p*=0.069). Third, men who were more positive in their attitudes toward using condoms tended to score higher on the eroticism from ejaculatory fluids scale than their counterparts who were more opposed to condom use (*r*=13, *p*=0.014).

Finally, none of the childhood maltreatment measures was found to be associated with semen arousal in the bivariate analyses.

The next step in the analysis was to subject the items identified above to a multivariate regression analysis. Table 3 presents the results of the items retained in the final, best fit model. As the table shows, five items were found to contribute uniquely and significantly to the prediction of the extent to which men eroticized ejaculatory fluids. These were: race (*p*=0.004), sexual role identity (*p*=0.039), educational attainment (*p*<0.001), HIV serostatus (*p*=0.040), and level of depression (*p*<0.001). Together, these items accounted for 12.2% of the total variance.

## Discussion

Before discussing the implications of the findings obtained in this study, the author wishes to acknowledge a few potential limitations of the research. First, as with most research data on sexual behaviors, the data in this study are based on uncorroborated self-reports. Therefore, it is unknown whether participants underreported or overreported their involvement in risky behaviors. The study’s reliance upon self-reported data is acceptable, however, as other authors of previous studies conducted with similar populations have reported good levels of data quality (e.g., reliability and validity) in their research [38]. This is particularly relevant for self-reported measures that involve relatively small occurrences (e.g., number of times having a particular kind of sex during the previous 30 days), which characterize the substantial majority of the data collected in this study [39]. Other researchers have also commented favorably on the reliability and/or the validity of self-reported information in their studies regarding topics such as condom use [40] and substance use/abuse [41,42].

A second potential limitation is the possibility of recall bias. For most of the measures used, respondents were asked about their beliefs, attitudes, and behaviors during the past 7 or 30 days. These time frames were chosen specifically: (1) to incorporate a large enough time frame in order to facilitate meaningful variability from person to person, and (2) to minimize recall bias. Although the authors cannot determine the exact extent to which recall bias affected the data, other researchers who have used similar measures have reported that recall bias is sufficiently minimal that its impact upon study findings is likely to be negligible [43]. This seems to be especially true when the recall period is small [44,45], as was the case for most of the main measures used in the present study.

Despite these potential limitations, the present study still yielded a number of interesting and important results. First, nearly one-half (41.9%) of the men who took part in this study reported ***overall*** semen arousal scores indicating a fairly high or high level of eroticism associated with various sensory aspects of giving or receiving semen. Perhaps a better indicator than their overall semen arousal scores, though, is a measure that takes into account whether the men reported being “fairly turned on” or “very turned on” by ***any*** of the sensory aspects of giving or receiving semen. When such a measure is developed, the data revealed that nearly two-thirds (65.1%) of the men who participated in *The Bareback Project* reported a high level of eroticism associated with at least one sensory aspect of giving or receiving semen. This is not terribly surprising when one considers the specific research population at hand–namely, men who use the Internet specifically to identify potential sex partners with whom they can engage in unprotected sex.

Nevertheless, this finding is noteworthy because it strongly suggests that one major reason why many MSM engage in unprotected sex is because they become aroused by the act of giving, receiving, and/or exchanging semen with their sex partners. For these men, engaging in unprotected sex involves more than merely the greater penile sensitivity and enhanced physical sensations associated with engaging in skin-to-skin sex compared with sex involving the use of a condom. For them, unprotected sex brings with it additional rewards, in the form of enjoying the way that the semen itself smells, feels, looks, and/or tastes. This aspect of HIV risk taking is one that, to the present author’s knowledge, very few previous or existing HIV prevention or intervention projects have addressed; yet it is a critical element of these risk taking behaviors–an element that must be considered and addressed squarely in prevention and intervention programs if progress is to be made with regard to lowering men’s risk of transmitting HIV.

The qualitative data from the study offer some interesting (and from an HIV prevention and intervention perspective, potentially valuable) insights into men’s mindsets when it comes to those who found the various sensory aspects of giving and/or receiving semen to be highly sexually arousing. Although the men who took part in *The Bareback Project* discussed numerous things that they liked about the way semen looks, feels, smells, and/or tastes, two themes emerged with a bit more frequency than others. Both of these themes deserve brief mentioning here, because both of them are topics that have been the subject of very little scientific discussion in the scholarly literature, and because both have potentially important implications when it comes to understanding the dynamics of semen arousal among bareback-sex-seeking MSM.

The first theme common to many (but certainly not all) of the men who found the various sensory aspects of giving and/or receiving semen to be highly arousing pertains to how men define themselves and their roles during the sexual act. In particular, many men who self-identified as sexual “tops” expressed the feeling that they considered it their duty or their job (i.e., their proper, expected role during sex) to provide semen to their sex partners. The interviewer’s post-interview notes for Respondent 815, a 54-year-old HIV-positive Caucasian man, are illuminating on this point: Respondent 815 is a 100% top, and he always ejaculates internally during anal sex. Even if men perform oral sex on him, he refuses to ejaculate anywhere other than inside of their anus. “I don’t use condoms, ever, and I don’t pull out, ever. Period. That’s just the way it is with me. That’s me being true to myself. Giving a guy my cum, or making him cum, means I’ve done what I was supposed to do. I did my job and I did it right.”

For Respondent 815 and others like him, the mere act of providing semen to his/their sex partners appears to be a source of satisfaction and reward, because their self-identities (at least in the sexual sense) are tied into their ability to produce and give semen.

The second theme that emerged from many of the interviews of the men who were highly aroused by the various sensory aspects of giving and/or receiving semen pertained to issues of control over the sexual proceedings, dominance of one’s sex partners, and creating deep-seated feelings of sexual desire, and how those phenomena intertwined. The interviewers’ notes for three participants in particular are informative on this point. Addressing this topic, the notes for Respondent 952, a 46-year-old HIV-positive Caucasian man, state: He does not like engaging in anal sex with anyone ejaculating inside of him, although he does like to do that as the insertive partner with HIV-positive men if they will beg him for his semen. The element of enhanced sexual desire, to the point of wanting to have his sex partners beg him for his semen, apparently played a large role in this man’s arousal by semen. As a second example, consider the comments made post-interview for Respondent 934, who was an HIV-negative 35-year-old Caucasian man: During the interview, he indicated that he would not be willing to withdraw from another man’s mouth or anus prior to ejaculation, even if asked to do so by the partner beforehand, until he has ejaculated at least one “shot” inside of the other person’s mouth or anus. If one of his partners wanted to withdraw from his mouth or anus before ejaculation, Respondent 934 said that he “would do everything in my power to bear down on him and keep him in place until he was finished shooting in me.” For this man (who definitely was *not* an isolated case in terms of expressing similar sentiments during the interview), a significant part of the attraction of the semen experience seems to have been the ability to control the sex and how the sex partner’s sexual behaviors “played out” during the sexual encounter. As a third and rather blunt example, there were copious notes taken in conjunction with the interview done with Respondent 986, a 26-year-old Caucasian HIV-positive man: He is a gift giver who self-identifies as a breeder. At the end of the interview he said that “I find it incredibly sensual to think of having my DNA inside of another man, who then can pass it on to other men for me.” Although he will have sex with any attractive man, he prefers to have sex with HIV-negative men who are eager to have him infect them with HIV. “The more they want my poz charged cum, the more I want to give it to them the more I need to give it to them.” Although this man is a “gift giver”–that is, an HIV-positive man who actively tries to find HIV-negative men whom he can infect with HIV–and although “gift givers” comprise only a small proportion of all barebacking MSM [46–48], Respondent 986’s comments are still consistent with the construct at hand–namely, becoming highly aroused by semen, at least in part, because of issues surrounding dominance and control.

Based on the present study’s findings, existing HIV prevention and intervention projects, as well as those that will be developed and implemented in the future, would be wise to include components that can “speak to” the various aspects of semen arousal and then work with gay and bisexual men to develop strategies to have their sexual needs met in a way that minimizes their risk for acquiring or transmitting HIV. This is likely to be easier said than done, however, as there are few functionally-equivalent alternative behavioral practices to suggest. After all, nothing truly is quite like semen in terms of appearance, consistency, flavor, and so forth. For men who are particularly aroused by the way that semen feels, one valuable and potentially feasible (i.e., palatable to the target community) approach might be to encourage these men to ejaculate onto their sex partners’ chests, necks, legs, or backs rather than inside of their mouths or anuses. For men who are especially aroused by the way that semen tastes, perhaps the best prevention/intervention message would be to suggest that these men allow semen to be accepted orally if they must, but not to swallow it. While this approach would not eliminate the risk of HIV transmission in such sexual situations, it would reduce this risk; and harm reduction is always a worthwhile goal to pursue when behavioral extinction is not perceived to be a viable option.

The preceding findings are all the more important to bear in mind and consider when developing future HIV prevention and intervention projects targeting MSM because of the present study’s findings that eroticism by semen was, oftentimes, related to actual HIV risk practices. As Table 2 shows, for three of the four HIV risk behavior outcome measures presented, men’s level of semen arousal was predictive of their involvement in the risk behavior in question even when the influence of other salient factors such as sexual role identity, HIV serostatus, substance use/abuse, sexual behavior preferences, and psychological/psychosocial functioning (among others) was taken into account. Unmistakably, semen arousal is an important measure to consider when evaluating men’s overall levels of HIV behavioral risk. Yet it is one that has, by and large, been ignored by previous researchers. In contrast, the link between substance use/abuse and HIV risk taking among MSM has been examined and discussed fairly widely [49–51]. Similarly, a few studies have devoted attention to the role played by psychological and psychosocial functioning and HIV risk practices among gay and bisexual men [38,52,53]. Many researchers have talked about the differences in HIV risk behavior involvement between HIV-positive and HIV-negative men [54,55]. But the extent to which gay and bisexual men are aroused by the various sensory aspects of giving, receiving, and/or exchanging semen has not been addressed sufficiently in the scientific literature; and this is one of the more unique aspects and contributions of the present study.

Additionally, as Table 3 portrays, the present research identified five factors that seem to be associated with greater semen arousal in this population of unprotected-sex-seeking, Internet-using MSM: belonging to a racial/ethnic group other than African American, self-identification as a sexual “bottom,” being better educated, being HIV-positive, and experiencing higher levels of depression. This is important to know, because it highlights the need for some targeted intervention and identifies just who it is who might benefit most from such intervention messages. The present study’s finding for sexual “tops” versus “bottoms” is consistent with some other MSM-focused studies [56,57] and highlights the need to provide targeted intervention with this group. Similarly, the present study’s finding that HIV-positive men were more aroused by semen (and that, in turn, placed them at greater risk for engaging in high-risk sexual behaviors) than their HIV-negative counterparts were is consistent with previous studies examining differences in HIV risk behavior practices among MSM based on their HIV serostatus [54,55] and with the published findings presented by Prestage, Hurley, and Brown in their discussion of the factors associated with so-called cum play. Again, it highlights the need for HIV-infected men to receive targeted intervention messages that can help them to lower their risk for transmitting HIV to their sex partners [14]. The present study’s findings pertaining to depression are also consistent with those reported by other researchers [58,59], once again indicating a need to provide targeted intervention for MSM who are suffering from this disorder. This particular issue has been addressed in much more detail by the present author in a separate work, and interested readers are encouraged to consult that article [60].

Finally, the author would like to emphasize and discuss one other intriguing finding derived by the present research–namely, that a not-inconsequential proportion of the study participants (30.4%) gave responses that indicated that, overall, they were not at all aroused or not very aroused by the various sensory aspects of giving or receiving semen. Yet these men, like the others who took part in *The Bareback Project*, actively sought unprotected sex online. It raises an important question: If they are not turned on by the way that semen feels or tastes or smells or looks, then why do they search for sex partners who are willing to give semen to them or receive semen from them, especially when the health risks of engaging in this practice are so high?

The qualitative data offer a few answers and insights here. First, for some of the men who were not aroused by the various sensory aspects of semen yet actively looked for partners for unprotected sex, self-identification as a sexual “top” seems to be a key factor. For these individuals, it is the way that they identify their role during sexual encounters and the meanings that they impute to that role that causes them to eschew condom use. The interview notes for Respondent 825, a 41-year-old African American man who is HIV-positive, are illustrative and informative on this point: “I am a total, domineering, controlling, no-pull-outs, horny black man topman, and I don’t ever change that for anybody!” He told me [the interviewer] that one main reason that he does not use condoms is that he cannot feel any sexual sensations when condoms are used on him, which causes him to lose his erection and, thus, be unable to ejaculate. “There’s nothing worse than having a bottom guy laying there saying ‘Fuck me and give me your load!’ and being unable to get it up to give it to him the way he wants and deserves.” he remarked.

Similarly informative are the interview notes for Respondent 953, a 53-year old HIV-positive man of multiracial descent: Perhaps most striking to me throughout the interview was his consistent adamancy about the fact that he has never used condoms and never will use condoms, with any partner under any circumstance. He said that he discloses his HIV serostatus to all of his partners before having sex with them so that they are aware that he is HIV-positive, and then engages exclusively in unprotected sex with them. He never discusses their sexual history or their

HIV status with them, because, as he put it “I tell them my status, and theirs doesn’t really matter to me after that. I try not to hurt anyone who comes into my life sexually, but I am a bareback top and I only bareback with other men I have sex with.” For these men (and others like them), arousal from semen is not a highly salient aspect of the decision to engage in unprotected sex; what they perceive to be their role as a “top” is more salient to their sexual decision making.

Second, a lack of being well-informed about HIV and how HIV is/not transmitted came up as a theme for some of the men who scored very low on the semen arousal measures yet still sought bareback sex partners. The post-interview notes for Respondent 542, a 36-year-old Caucasian man who is HIV-infected, illustrate this point: the most striking part of this interview was this respondent’s inaccurate beliefs about HIV and HIV transmission. Most notably, he reported thinking that there is a cure for AIDS, a vaccine to prevent HIV, and that men cannot get infected if their partner withdraws prior to ejaculation. Likewise, the interviewer’s notes for Respondent 953, a 53-year-old HIV-positive man of multiracial descent, also addressed this point: He said that he had no idea whether or not there is a cure for AIDS, and he was unsure about whether or not there is a vaccine to prevent HIV infection. He guessed at his answers to 6 of the 15 questions, leaving him a correct response score of only 7 questions. Presumably, at least to some extent, the desire to engage in unprotected sex can be attributed to a lack of understanding of just how it is that HIV is transmitted from person to person.

Third, indifference as to whether or not they become infected with HIV (if they were HIV-negative) or whether or not they infect their sex partners with HIV (if they were HIV-positive) was another theme amongst several of the respondents who scored low on the semen arousal measures. Such was the case for Respondent 542, a 36-year-old Caucasian man who is HIV-positive, and Respondent 849, a 26-year-old HIV-negative African American man, whose interviewer’s notes indicate: He never uses condoms with any of his sex partners, and does not want them to use condoms with him. He thinks that he had a 50-50 chance of becoming HIV-infected, but considers it only somewhat important that he remains HIV-negative. For these men, engaging in unprotected sex simply appears to be something that they do, without regard or great concern for the consequences, and is not “driven” by a desire or a lack of desire for giving or receiving or exchanging semen.

Fourth, having an overt dislike for condoms regardless of the consequences of refusing to use them was another commonality amongst several of the men who scored very low on the semen arousal measures. In this context, sometimes the men had reasons for disliking condoms, and in other instances, they expressed no specific reasons for disliking them. For example, Respondent 006, a 60-year-old HIV-positive Caucasian man commented that using condoms during sex “would be like putting a bag over your head while trying to eat your favorite food.” As another example, consider the interviewer’s notes for Respondent 939, an HIV-positive 37-year-old Latino man: He will allow a partner to use them [condoms] if the partner wishes, but prefers this not to be the case. He will not broach the subject of condoms with his partners and said over and over and over again throughout the interview how much he hates them, dislikes them, won’t use them, and doesn’t even want to think about them.

Ultimately, however, despite these contributory explanations as to why some men who do not find semen to be arousing yet still seek unprotected sex, we are left with a need to examine this issue more completely. Although such individuals did not comprise the majority of the participants in this study, their presence was not negligible and their behaviors merit a much better understanding than we currently possess. This, along with additional efforts to understand more about the meanings that exist in the overall framework of giving, receiving, and exchanging semen with one’s sex partners, would be a fruitful avenue for future researchers to pursue.

## Figures and Tables

**Table 1:** Semen arousal among *bareback project* study participants.

Extent of Semen Arousal	Aroused by Taste (%)	Aroused by Smell (%)	Aroused by Feel (%)	Aroused by Sight (%)	Overall Arousal (%)
**Not turned on at all**	18.7	29.2	19.3	8.1	10.5
**Not turned on very much**	15.4	18.1	10.8	7.5	19.9
**Somewhat turned on**	24.8	22.3	18.1	20.8	27.7
**Fairly turned on**	12.7	8.4	15.8	18.5	25.6
**Very turned on**	28.4	22.0	37.1	46.1	16.3

**Table 2:** Multivariate results* for the relevance of eroticizing ejaculatory fluids, for four selected sexual risk measures.

Independent Variable	Proportion of Sex Acts Involving Internal Ejaculation	Proportion of Oral Sex Acts Involving Internal Ejaculation	Any Involvement in Multiple-Partner Sex?	Any Involvement in Anonymous Sex?
Eroticizing ejaculatory fluids	0.15 (0.008)	0.21 (<0.001)	0.21 (0.005)	0.11 (0.095)
Sexual role identity=bottom	0.16 (0.006)	0.13 (0.019)	———	———
Sexual orientation=gay	0.13 (0.022)	———	———	———
HIV serostatus=HIV-positive	———	———	0.15 (0.045)	0.16 (0.021)
Any recent methamphetamine use	———	———	0.30 (0.002)	———
Number of type of illegal drugs used	———	———	0.27 (0.004)	———
Any recent illegal drug use	———	———	———	0.18 (0.007)
Preference for sex that is rough	———	———	0.19 (0.013)	———
Preference for sex in public places	———	———	———	0.16 (0.031)
Perceived accuracy of HIV serostatus information provided in online profiles	———	———	0.16 (0.032)	———
Average number of hours spent per day searching for sex partners online	———	———	0.17 (0.032)	———
HIV/AIDS knowledge quiz score=100%	———	———	———	0.18 (0.007)
HIV information burnout	———	———	———	0.24 (0.001)
Partner communication skills	———	———	0.16 (0.031)	———
R-squared**	**0.074**	**0.069**	**0.326**	**0.193**

* The figures presented in this table indicate standardized coefficients (i.e., beta values), to facilitate comparisons of effects sizes from measure to measure. Beneath each coefficient, in parentheses, is the level of statistical significance for that coefficient.

** Multivariate logistic regression was used for the two right-hand columns. Therefore, the R^2^ values shown for those equations are pseudo-R^2^ values, as a true R^2^ value is not computed in conjunction with this statistical technique.

**Table 3:** Multivariate predictors of semen arousal.

Independent Variable	Standardized Coefficient (β)	*p*=|x|
Race=African American	−0.15	0.004
Sexual role identity=bottom	0.11	0.039
Education attainment=high school diploma or less	−0.19	<0.001
HIV serostatus=HIV-positive	0.11	0.040
Level of depression	0.18	<0.001
**R-squared**	**0.122**	
